# Genetic Factors in Systemic Lupus Erythematosus: Contribution to Disease Phenotype

**DOI:** 10.1155/2015/745647

**Published:** 2015-12-21

**Authors:** Fulvia Ceccarelli, Carlo Perricone, Paola Borgiani, Cinzia Ciccacci, Sara Rufini, Enrica Cipriano, Cristiano Alessandri, Francesca Romana Spinelli, Antonio Sili Scavalli, Giuseppe Novelli, Guido Valesini, Fabrizio Conti

**Affiliations:** ^1^Reumatologia, Dipartimento di Medicina Interna e Specialità Mediche, Sapienza Università di Roma, 00161 Rome, Italy; ^2^Department of Biomedicine and Prevention, Section of Genetics, School of Medicine, University of Rome “Tor Vergata”, 00133 Rome, Italy

## Abstract

Genetic factors exert an important role in determining Systemic Lupus Erythematosus (SLE) susceptibility, interplaying with environmental factors. Several genetic studies in various SLE populations have identified numerous susceptibility loci. From a clinical point of view, SLE is characterized by a great heterogeneity in terms of clinical and laboratory manifestations. As widely demonstrated, specific laboratory features are associated with clinical disease subset, with different severity degree. Similarly, in the last years, an association between specific phenotypes and genetic variants has been identified, allowing the possibility to elucidate different mechanisms and pathways accountable for disease manifestations. However, except for Lupus Nephritis (LN), no studies have been designed to identify the genetic variants associated with the development of different phenotypes. In this review, we will report data currently known about this specific association.

## 1. Introduction

Systemic Lupus Erythematosus (SLE) is an autoimmune disease with multifactorial etiology, in which genetic and environmental factors interplay determining disease susceptibility [1].

**Figure 1:**
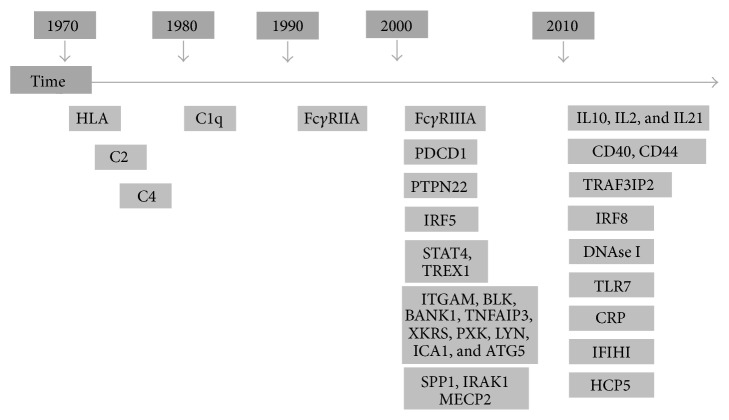
Schematic representation of genetic variants associated with SLE susceptibility identified from 1970.

Starting from 1970, several genetic studies in various SLE populations have identified numerous susceptibility loci. However, the genetic variability so far identified accounts for less than half of the SLE heritability, with modest overall effect sizes (OR ~ 1.5 to 1.2) (Figure 1) [2–7]. It is well established that some specific genetic factors are not shared between all SLE patients, excluding a role in the disease susceptibility and suggesting an association with specific phenotypes (Table 1) [6, 8]. However, this discrepancy could be related to multiple mechanisms that can lead to SLE development.

As widely demonstrated, specific autoantibodies resulted in being associated with different disease-related manifestations, identifying distinctive subset in terms of morbidity and mortality and suggesting different underlying etiologies [9]. Similarly, in the last years, some studies have evaluated the relationships between SLE risk genes and disease phenotypes, in order to elucidate different mechanisms and pathways accountable for disease manifestations. However, except for Lupus Nephritis (LN), no studies have been specifically designed to evaluate the genetic risk factors associated with different manifestations. Therefore, these data could be extrapolated from studies evaluating disease susceptibility, which include a genotype-phenotype analysis.

## 2. Renal Involvement 

Renal involvement could affect up to 60% of SLE patients, as initial manifestation or during disease course. Despite the improvement in terms of diagnostic accuracy and management, LN patients showed higher morbidity and mortality compared with those without this manifestation [10]. Accordingly, the identification of markers able to identify most severe disease and to predict the end-stage renal disease (ESRD) development is a crucial topic. In particular, during the last years, numerous attempts have been made in order to identify serological and urinary biomarkers able to discriminate the different severity degree and to monitor response to treatment in LN patients, obtaining contrasting results [11]. Moreover, the use of resistive index (RI) as a severity marker in LN patients has been suggested in a recent study published by Conti et al. The authors identified a significant association between a pathologic RI (>0.7) and class IV glomerulonephritis, widely identified as the most severe [12].

In the context of biomarkers, genetic factors could have an important role in SLE patients with renal involvement in order to identify subject at risk to develop most severe and rapidly progressive forms. Moving from the genetic variants previously associated with disease susceptibility, several studies have verified the association of the same alleles with the presence of renal involvement.

The first genetic association described for SLE based on the case-control methodology was with the human leucocyte antigen (HLA) region at chromosome 6p21.3, encoding more than 200 genes, many of them with a specific immunological role. Seven HLA Class II alleles were demonstrated to be significantly associated with SLE and LN [13]. The HLA-DR2 and DR3 alleles resulted in being the most strongly associated with SLE susceptibility in African, Asian, European, and North, Central, and South American populations, even though HLA-DR3 tends to be more associated in European-derived populations [8]. In particular, the association between the disease susceptibility and highly conserved HLA-DRB1^*∗*^03:01 and HLA-DRB1^*∗*^15:01 haplotypes has been well established in European populations [8]. The punctual mechanism by which HLA-DR alleles determine an increased risk to develop SLE is not completely defined. The most reliable hypothesis suggests the influence of HLA-DR on the selection and enrichment of autoreactive T cells through the presentation of molecular mimics [14].

Moving from these premises, the association with renal involvement has been investigated, showing the primary role exerted by HLA-DR3 and DR-2 [15]. In particular, the results obtained from the study conducted by Taylor and colleagues in 2011 and by Bolin in 2013 showed the association between HLA-DR3 and LN (OR = 1.37 and *P* < 1 × 10^−4^, resp.) [16, 17]. Particularly, Bolin et al. found an association between HLA-DR3 allele and proliferative nephritis (*P* < 0.001) [17].

Numerous evidences identified an association between* signal transducer and activator of transcription 4* (STAT4) genetic variants and increased risk to develop SLE, suggesting a role of these genetic variants in influencing disease phenotype.

In 2008, Taylor and colleagues analyzed a large SLE population, obtained from four sources (UCSF Lupus Genetics Project; Autoimmune Biomarkers Collaborative Network; Multiple Autoimmune Disease Genetics Consortium; Pittsburgh Lupus Registry) in order to evaluate the association between SNP rs7574865 of STAT4 and the different SLE-related manifestations. The phenotype case-only analysis identified a significant association with the presence of severe nephritis, defined as ESRD, or histopathologic evidence of severe, progressive renal disease (OR = 1.50) [18]. Simultaneously, the study conducted by Kawasaki et al. in a Japanese population confirmed the association between this STAT4 risk allele and renal involvement. In particular, the authors identified the association with rs7574865 both in SLE patients with nephritis (OR = 1.85) and in those without (OR = 1.55), which was stronger in nephritis cases [19].

More recently, Bolin and colleagues evaluated a cohort constituted by 567 Swedish Caucasian SLE patients and 512 healthy controls to elucidate the genetic components of LN [17]. By performing a LN case/controls analysis, a significant association between the SNPs rs11889341, rs7574865, rs7568275, and rs7582694 of STAT4 gene and the development of LN (*r* ^2^ = 0.98) was identified. In particular, the rs7582694 allele resulted in being associated with the presence of a proliferative nephritis (OR = 2.27) and with the development of a severe renal insufficiency (defined as a GRF < 30 mL/min/1.73 m^2^ at follow-up) (OR = 3.61) [17]. This association was not always confirmed. In the study conducted by Li and colleagues in 2011 on a Northern Han Chinese SLE population, the SNP rs7574865 of STAT4 did not show any correlation with clinical manifestations [20].

Several hypothesis have been suggested to understand the mechanisms by which STAT4 could contribute to LN development. Interleukin-12 (IL-12), the main STAT4 activating cytokine, is able to induce the Th1 and Th17 differentiation with consequent production of IFN-*γ* and IL-17. These specific pathways seem to be crucial for the LN pathogenic mechanism: in particular, IL-17 could exert a direct role, as demonstrated by the identification of Th17 cells in kidney tissue and by the association between high IL-17 levels and less favorable outcome [21]. Moreover, SLE patients carrying the STAT4 risk allele rs7574865 showed an increased sensitivity to IFN-*α* signaling, as demonstrated by the overexpression of IFN-*α* regulated gene. Among these, TNFSF13B codifies the B lymphocyte stimulator (BLyS), which is able to promote B cell differentiation and autoantibody production [22].

The influence of ITGAM genetic variants on SLE susceptibility has been demonstrated in populations with different ethnicity: in particular, convincing data derived from European ancestry, but also from Hispanic, African-Americans, Mexicans, and Colombians cohorts [23]. Moving from these results, the association with specific clinical manifestations has been investigated. In 2009, Yang and colleagues identified a significant association between renal involvement and the ITGAM risk alleles rs1143683 and rs1143679 (OR = 3.35, OR = 2.05, resp.) in a Hong Kong SLE cohort [24]. Moreover, this association was confirmed in an analysis conducted on Finnish and Swedish population of patients affected by SLE. The authors observed increased risk in SLE patients with renal involvement, with an OR = 2.49 for the rs1143679 SNP [25].

The study conducted by Kim-Howard and colleagues in 2010 on a very large population, constituted by 2366 SLE patients and 2931 unaffected controls with European ancestry, confirmed the link between the genetic variant rs1143679 of ITGAM and renal disorders as defined by the American College of Rheumatology (ACR) criteria (OR = 1.39) [26]. To better assess the magnitude of this association, a comparison between patients with the specific ACR-criteria renal manifestations and healthy controls was performed. This statistical approach allowed the identification of a strong effect concerning the association with renal criteria (OR = 2.15) [26].

More recently, in 2011 Sanchez et al. confirmed this association (OR = 1.25) by evaluating a population constituted by 4001 European-derived, 1547 Hispanic, 1590 African-American, and 1191 Asian patients. This association seems to be driven prevalently by the European-derived cohort, as demonstrated by a higher OR in this specific subset (OR = 1.39) and by the lack of a significant association with African-American or Asian individuals [27].

ITGAM encodes the CD11b chain of the Mac-1 (alphaMbeta2; CD11b/CD18; complement receptor-3) integrin, a surface receptor protein implicated in the interaction of monocytes, macrophages, and granulocytes. The genetic variants of this molecule, resulting in amino acid substitution in the extracellular portion, could determine a dysfunctional integrin, not able to mediate cell adhesion to integrin ligands and phagocytosis. Moreover, this defective integrin does not seem to be able to restrict the production of inflammatory cytokines in macrophages [28].

Under physiological conditions, ITGAM is expressed by endothelial cells of glomerular and peritubular capillaries of Bowman's capsule. It has been suggested that an increased expression of a defective molecule, due to the presence of a genetic variant, could be associated with a loss of clearance of glomerular deposits, with inflammatory process development [26]. Similarly, defective handling of immune complexes could be the mechanism explaining the association between genetic variants of FCGR3A and kidney involvement in SLE patients. A meta-analysis conducted by Karassa and colleagues in 2003 evaluated the study examining the association of the FCGR3A V/F158 polymorphism and LN, published until August 2002 [29]. Data deriving from the analysis on 16 studies demonstrated a significant overrepresentation of the low-binding F158 allele in patients with renal disease compared with those without (*P* = 0.003). Moreover, the presence of this allele seems to confer a 1.2-fold greater risk for renal disease development, irrespective of the ethnicity [29]. More recently, the PROFILE cohort, constituted by 1008 SLE patients, with renal involvement in 43.4% of the cases (438 patients), was evaluated in order to identify the association with FCGR3A polymorphism. The authors identified an overrepresentation of FCGR3A^*∗*^GG in SLE patient developing ESRD (21.9%) compared with those who did not develop it (7.5%) (*P* = 0.0175) [30]. Interestingly, the evaluation of FCGR3A variants demonstrated different genetic association for the global lupus phenotype and for the renal involvement (FCGR3A^*∗*^T and FCGR3A^*∗*^GG, resp.). This observation confirms the hypothesis of different genetic background for susceptibility and disease phenotype, leading to different pathogenic mechanisms associated with the corresponding molecule [30].

FCGR plays a pivotal role in removing antigen-antibody complexes at the tissue and organ level. Allelic variants could alter this function, causing an inflammatory response with damage development. In particular, as widely demonstrated, a homozygosity condition for this FCGR3A SNP could lead to impaired handling of immune complexes, causing a proinflammatory status [29, 30]. This could justify the impact of genetic variants of FCGR3A in the determination of a specific phenotype, such as renal involvement.

In the last 20 years, the role of Interferon (IFN) signature in the SLE pathogenesis has been recognized, as demonstrated by the dysregulation in the expression of genes in the IFN pathway in more than half of SLE patients [31]. IFN pathway is involved in several pathologic mechanisms, involving Th1 and B cells activation and survival. Moreover, IFN acts as a bridge between innate and adaptive immune systems. Interferon Regulatory Factors (IRF) ensure the regulation of this complex pathway, by acting on signaling and immune cell development [31]. Genetic variants of IRF5, IRF7, and IRF8 genes have been associated with SLE susceptibility (OR = 1.88, OR = 0.78, and OR = 1.17, resp.) since they associated with increased levels of protein expression [32]. Starting from these evidences, the association with renal involvement in SLE patients has been investigated. The study conducted on 190 LN patients and 182 healthy Chinese blood donors demonstrated a significantly higher frequency of the T allele of IRF5 rs2004640 SNP in LN patients (OR = 1.60) [33].

The abovementioned study conducted by Bolin and colleagues in 2013 identified a strong association between LN and two nearly perfectly linked SNPs in IRF5 (rs2070197 and rs10488631, *r* ^2^ 1.0). In particular, the risk allele C of rs10488631 was associated with proliferative nephritis (OR = 2.61) and severe renal insufficiency (OR = 3.03) [17]. More recently, an association between IRF7 rs4963128 and LN (OR = 2.69) has been identified in the study conducted by Li and colleagues in 2011 in a Northern Han Chinese population [34].

In 2011, for the first time, Sanchez and colleagues suggested a new interesting genetic factor related to renal disorders in SLE patients by identifying a significant association with rs2205960 TNFSF4 risk allele (OR = 1.14) [27]. TNFSF4, also called OX40L, is a member of the TNF superfamily, expressed prevalently on antigen-presenting cells; activated T cells express the receptor of this molecule (TNFSFR4 or OX40) [35]. The expression of TNFSF4 at the epithelial level of the glomerular capillary has been demonstrated in LN patients [36]. More recently, Zhou and colleagues demonstrated a modification of cytokine production in PBMC in LN patients after treatment with anti-CD134 monoclonal antibody [37]. Finally, significantly higher TNFSF4 serum levels have been demonstrated in SLE patients with renal involvement, compared with patients without nephritis, suggesting the role of this molecule as a marker. Moreover, the increased expression on CD4 positive T cells seems to be associated with LN and disease activity [38]. Taken together, these evidences could justify the link between a genetic variant in the TNFSF4 gene and renal involvement.

Several other genetic variants have been associated with kidney manifestations in SLE patients. Panneer and colleagues suggested the role of polymorphism in the gene codifying the DNAse I, an endonuclease involved in the cleavage and clearance of chromatin during apoptotic processes [39]. The reduction of the DNAse I function, related to the genetic modification, could alter this cleavage and the clearance of immune-complexes and NETs, resulting in the persistence of apoptotic debris [40]. By evaluating 300 South Indian Tamil SLE patients, the authors identified a significantly higher frequency of heterozygous genotype of Q222R polymorphism in patients with nephritis than in those without (67% versus 53%, OR = 1.93) [39]. Some interesting data concerning the association between LN development and polymorphism on the gene codifying C1q have been recently published [41, 42]. However, due to the small cohorts evaluated in these studies, their results should be confirmed in large populations.

## 3. Neuropsychiatric Manifestations

Neuropsychiatric SLE is a major disease manifestation, characterized by a wide heterogeneity in terms of clinical features, degrees of morbidity, and severity between patients [43]. A percentage of SLE patients ranging from 14 to 75% may refer to neurological symptoms: this wide variability is probably related to the great heterogeneity of this disease manifestation. Despite the relevance of this involvement, studies focusing on genetic variants specifically associated with NPSLE have been rarely conducted. Nonetheless, Koga and colleagues in 2011 evaluated 282 Japanese SLE patients and 222 healthy controls in order to assess the cumulative number of risk alleles associated with SNPs of HLA-DRB1, IRF5, STAT4, BLK, TNFAIP3, TNIP1, FCGR2B, and TNFSF13 genes. SLE patients registered a significantly higher number of risk alleles compared with controls (8.07 ± 1.60* versus *7.02 ± 1.64, *P* = 1.63 × 10^−12^). Interestingly, when considering SLE patients carrying more than 10 risk alleles, the proportion of patients with neurological involvement was significantly higher compared with subjects with a number of risk alleles lower than 10 (OR = 2.30). This result could suggest that a higher number of risk alleles could determine most severe disease manifestations [44].

Genetic variants in TREX1 gene, codifying a three­prime repair exonuclease 1 (also known as DNAse III), have been considered a good candidate for NPSLE. de Vries and colleagues scanned genomic DNA of 60 NPSLE patients for exonic TREX1 mutations using direct sequencing. This study identified a novel heterozygous p.Arg128His mutation in one NPSLE patient, admitted to the hospital because of lethargy and progressive migraine-like headache [45]. The authors suggested that the p.Arg128His mutation is responsible for neurological manifestations at the light of the absence of this mutation in 400 control chromosomes and in 1712 healthy individuals, previously screened by Lee-Kirsch et al. [46]. More recently, this association has been confirmed in the study conducted by Namjou and colleagues in 2011. By evaluating the European population enrolled in the analysis, the authors identified a significant association between the presence of neurological manifestations (as defined by ACR criteria), especially seizure, and specific variants in TREX1 gene. In particular, the rs922075 (OR = 1.644); rs6776700 (OR = 1.689); rs6442123 (OR = 1.747); rs2242150 (OR = 1.638); rs11797 (OR = 1.714) SNPs resulted in being significantly associated [47].

## 4. Joint Involvement

Joint involvement is a frequent manifestation in patients with SLE and could affect up to 90% of patients. A wide heterogeneity, varying from arthralgia to erosive arthritis similar to rheumatoid arthritis, characterizes this manifestation [48]. Nevertheless, the number of reports is relatively scarce. Concerning the identification of specific genetic variants, few studies have evaluated the association with joint involvement.

ITGAM gene risk variants have been associated with arthritis in SLE patients. The study conducted by Warchoł et al. in 2011 in a Polish SLE population demonstrated an association between the rs1143679 genetic variant and occurrence of arthritis (OR = 3.486) [49].

A strong association with arthritis and Vitamin D Receptor (VDR) polymorphism was identified in the study conducted by de Azevêdo Silva and colleagues in 2013 [50]. Through the Vitamin D Receptor (VDR), Vitamin D exerts an immune-modulatory effect. In particular, it intervenes in downregulation of Th1 immune response, modulation of dendritic cells differentiation, depressing activated B cell proliferation, upregulation of regulatory T cells, and preserving immune response [50]. A number of evidences showed that patients with SLE often present reduced levels of Vitamin D suggesting an involvement of this molecule in disease pathogenesis [51]. There is still a debate concerning the precise role of VDR in SLE [52]. The study conducted by de Azevêdo Silva in 2013 did not identify any association between VDR polymorphism and SLE susceptibility. Conversely, the T/T genotype (rs3890733) resulted in being significantly associated with the presence of joint involvement (OR = 17.05). The authors underlined that this association should be interpreted with caution because the frequencies observed for this VDR polymorphism were not in Hardy-Weinberg equilibrium [50].

Other associations between genetic variants and joint involvement have been suggested: some data identified an association with C4 and ACP5 genetic variants, but no replication studies are available [53, 54]. Moreover, Ciccacci and colleagues identified an association between joint involvement and rs2910164 of mir146a gene (OR = 1.93) [55].

The association between arthritis and the FCGR2A and FCGR3A low copy number genotype has been identified in a cohort of Taiwan SLE patients [56, 57]. In particular, in the most recent study, the FCGR3A low copy number genotype was significantly enriched in SLE patients with arthritis (*P* = 0.001; OR = 1.56) [57].

Finally, the study conducted by Fonseca et al. in 2013 identified an association between arthritis and the SNP rs15866 of STK17A gene (OR = 2.92), encoding serine/threonine-protein kinase 17A [58]. The mechanism explaining this association is not clarified and replication studies are needed to confirm these results.

## 5. Skin Manifestations

Skin involvement represents a frequent manifestation in SLE patients (up to 75%), characterized by a great heterogeneity, including acute and chronic phenotypes. Some genetic variants have been associated with different skin manifestations in SLE cohorts.

ITGAM genetic polymorphisms are to date the most frequently associated variants with skin involvement. In 2010, Kim-Howard et al. have identified an association between malar rash and the polymorphism rs1143679 of ITGAM (OR = 1.27) [26]. Moreover, the presence of discoid rash resulted in being associated with ITGAM rs1143679 (OR = 1.20) in the study conducted by Sanchez et al. in 2011 [27].

Järvinen and colleagues conducted an analysis specifically designed to address the role of ITGAM genetic variants in a cohort of Finnish and Swedish patients with discoid LE, without signs of systemic disease. The analysis demonstrated a strong association between the allele rs1143679 and DLE (OR = 3.2). The authors identified a significant association also in SLE patients with discoid rash (OR = 3.76). Moreover, other variants in ITGAM resulted in being associated with these manifestations, but the authors hypothesized that the strong linkage disequilibrium with rs1143679 could explain this result [25].

The link between ITGAM and photosensitivity, frequently identified in patients with discoid LE, could explain this association. Ultraviolet- (UV-) B irradiation determines the activation of several proinflammatory events at the skin level, involving prevalently macrophages ITGAM-expressing and dendritic cells. Genetic-determined modification in the function of ITGAM could modify the processes regulating the dendritic cell differentiation, inducing inflammatory reactions in discoid LE patients [25]. On the other hand, the absence of CD11b seems to enhance the differentiation of naive T cells to IL-17 producing Th17 cells, determining the increase of IL-17 serum levels, identified in discoid LE and SLE patients with skin involvement [59].

Moreover, genetic variants of FCGR2A seem to be associated with skin manifestations. In particular, Sanchez and colleagues identified an association between malar rash and FCGR2A rs1801274 (OR = 1.11) [27].

The abovementioned study conducted by de Azevêdo Silva et al. in 2013 identified an association between the SNP rs11168268 of VDR and cutaneous alterations in a cohort of Brazilian SLE patients [50]. Photosensitivity, one of the most common cutaneous alterations described in SLE patients derives from the exposure to UV light, causing a macular or erythematous rash. After UV exposure, keratinocytes begin apoptotic process due to DNA damage with release of nuclear material. A defective clearance of apoptotic body could trigger an immune response. Vitamin D has proved to be able to reduce the UV-induced DNA damage and suppress cutaneous immunity, playing an important role in the maintenance of cell integrity after UV light exposure [60]. The presence of genetic variants in VDR, expressed in the skin epithelial cells, could modify this Vitamin D ability, promoting cutaneous alterations in SLE patients [61].

A recent meta-analysis identified a significant association between the IL-6-174 G/C polymorphism and discoid skin lesions by the evaluation of 15 studies (OR = 2.271). These results support the role of IL-6 in the pathogenesis discoid skin lesions [62].

## 6. Serositis

Few data are available in the literature about the genetic risk for the serositis development. The study published by Perricone et al. in 2013 identified an interesting correlation between the TRAF3IP2 SNPs and the development of pericarditis. The authors identified a significant association with the three TRAF3IP2 SNPs evaluated (rs33980500: OR = 2.59; rs13190932: OR = 2.38; rs13196377: OR = 2.44). Moreover, the authors analyzed the contribution of SLE antibody to the development of this specific manifestation, showing a significant association between the risk to develop pericarditis and anti-La/SSB positivity (OR = 2.65). A binary logistic regression analysis demonstrated that both TRAF3IP2 rs33980500 and anti-La/SSB could be independently associated with the development of pericarditis (*P* = 0.006 and *P* = 0.032, resp.) [63]. In this study, for the first time, the role of TRAF3IP2 genetic variants on SLE susceptibility has been ascertained. Interestingly, TRAF3IP2 polymorphism resulted also in being associated with a specific disease manifestation. TRAF3IP2 codifies the molecule Act1, which from one side is involved in the IL-17 pathways, but it is also a negative regulator of the CD40-mediated signaling pathway [64, 65].

The study conducted by Ciccacci and colleagues in 2014 identified a new association between the occurrence of pericarditis and the genetic variant rs2542151 of PTPN2 gene (OR = 2.49) [55]. PTPN2 codifies the enzyme tyrosine-protein phosphatase nonreceptor type 2, a member of the protein tyrosine kinases (PTP) superfamily. PTPN2 genetic variants have been previously associated with susceptibility to both Crohn's disease and ulcerative colitis and with an earlier onset of type 1 diabetes [66, 67]. The abovementioned study by Ciccacci and colleagues analyzed for the first time the role of PTPN2 genetic variants in the SLE susceptibility, without identifying significant differences between patients and healthy controls. Conversely, the SNP rs2542151 of PTPN2 resulted in being associated with serositis, and specifically with pericarditis [55]. The relevance of this association should be clarified by larger studies.

## 7. Hematological Manifestations

Similarly to the other SLE-related manifestations, few studies focusing on the association between genetic variants and hematological features have been conducted to date. The extrapolation from genotype-phenotype studies identified some associations. Among these, Sanchez and colleagues in 2011 identified an association between hematological features and IL-21 rs907715 (OR = 1.13). When the different ACR hematological criteria were analyzed, an association with leucopenia was confirmed (OR = 1.14). IL-21, primarily produced by activated CD4+ T cells, is involved in differentiation and functional activity of T and B cells [68–70]. This evidence could justify this association, by hypothesizing that a genetic variant of IL-21 could be related to a modification of this activity on B and T cells, influencing disease phenotype.

More recently, Fonseca et al. in 2013 described a significant association between hematological features and haplotype TAGTC of STK17A gene (OR = 0.03). The patients stratification according to ethnicity and gender suggested a protective role of this haplotype on hematological manifestations development (OR 0.37) [58]. Similarly to the association with arthritis, the mechanism explaining this association is not identified and other replication studies are needed to confirm these results.

## 8. Immunological Abnormalities

The production of a wide range of autoantibodies, resulting from polyclonal B cells activation, impaired apoptotic pathways, or idiotypic network dysregulation, characterizes SLE [1, 71]. Among these, the anti-double-stranded DNA antibodies (anti-dsDNA) are considered the most specific marker for SLE, due to their high frequency (ranging from 70% to 98%) and sensitivity and specificity (57.3% and 97.4%, resp.) [72, 73].

Several evidences suggested a role of genetic factors in autoantibodies determination [74]. The same genetic variants, previously described as associated with renal involvement, have been investigated in order to identify a link with anti-dsDNA production. Since 1998, Podrebarac and colleagues described the association between anti-dsDNA production and the presence of HLA-DRB1^*∗*^1501 (DR2) allele [75]. More recently, the association between HLA-DR2 and DR3 with the presence of anti-dsDNA has been confirmed by several analysis [16, 76].

Starting from 2008, the association between the STAT4-risk allele of the SNP rs7582694 and positivity for anti-dsDNA has been identified by different studies [16, 18, 77]. Finally, ITGAM polymorphism has been also associated with the presence of anti-dsDNA. In particular, the study conducted by Kim-Howard and colleagues in 2010 identified an association with rs1143679 (OR 1.65) in a case-only analysis performed by comparing SLE patients positive and negative for anti-dsDNA [26].

Four years ago, Chung and colleagues conducted the first genome wide study focused to identify genetic factors associated with anti-dsDNA autoantibody production, by analyzing 1278 SLE cases and 3334 healthy controls of European descent [78]. Genetic variants STAT4 (rs7574865), IRF5 (rs10488631), ITGAM (rs9888739), and MHC (HLA-DR3, rs2187668) resulted in being strongly associated with anti-dsDNA positivity (OR = 1.77, OR = 1.92, OR = 1.80, and OR = 2.23, resp.). Moreover, the authors assessed the relationship between the anti-dsDNA autoantibody production and the cumulative genetic risk, calculated by counting the total number of risk alleles identified in a single subject. The mean SLE genetic risk was higher in SLE patients positive for anti-dsDNA (15.5 ± 3.1) compared with anti-dsDNA negative patients (14.5 ± 3.0) and healthy controls (13.1 ± 2.8), even though this difference was not significant [78]. The results of this study suggest that some genetic variants are more strongly associated with anti-dsDNA autoantibody production than with SLE susceptibility, and they could be described as “autoantibody propensity genes” [78].

Even though the majority of the studies have focused on the anti-dsDNA antibodies, some evidences demonstrated an association between genetic variants and production of other autoantibodies in patients affected by SLE.

Järvinen and colleagues in 2010 identified an association between the polymorphism rs1143679 in ITGAM gene and the presence of Ro/SSA autoantibodies in the Finnish (OR = 2.65) and Swedish (OR 1.62) populations [25]. The involvement of both Ro-autoantibodies and the ITGAM protein product in the same biological pathways of apoptosis and phagocytosis could explain this association, which remains mostly unknown [25].

The study conducted by Li and colleagues in 2011 suggested a new association between IRF7 rs4963128 polymorphism and anti-SSA/SSB (OR = 0.61) [20]. Moreover, the study conducted by Ciccacci and colleagues in 2014 identified for the first time an association between anti-Ro/SSA and HCP5 rs3099855 polymorphism (OR = 2.28) [55]. This SNP has been previously associated not only with Steven Johnson syndrome and toxic epidermal necrolysis susceptibility, but also with primary sclerosing cholangitis, another autoimmune condition [79, 80]. More interestingly, as demonstrated by a genome-wide association study, the same polymorphism resulted in being associated with cardiac manifestations of SLE, a clinical condition frequently associated with the presence of anti-Ro/SSA antibodies [81]. These data suggest a pathological link between anti-Ro/SSA antibodies and this HCP5 polymorphism, requiring further studies to clarify the specific underlying mechanisms. Moreover, both anti-Ro/SSA and anti-La/SSB autoantibodies resulted in being significantly associated with HLA-DRB1^*∗*^03:01 (OR = 1.60, OR = 2.57, resp.), as demonstrated by the largest SLE subphenotype genetic association study conducted so far [82].

A recent study published by Niewold et al. in 2012 evaluated the association between IFR5 haplotype and different SLE-related manifestations. Interestingly, the authors identified a strong and strikingly distinct association between different autoantibodies and different IRF5 haplotypes. In particular, TACA haplotype was associated with anti-dsDNA and anti-Ro/SSA (OR = 1.5, OR = 1.51, resp.), TATA haplotype with anti-dsDNA (OR = 1.68), and TCTA haplotype with anti-La/SSB (OR = 3.51) [83]. These results suggest the possible role of IRF5 genotype to predispose the antibodies formation: IRF5 haplotypes could influence susceptibility to form particular antibodies. Immune complexes containing these antibodies are internalized into cells, and the nucleic acid component could trigger endosomal TLR7 and TLR9. The presence of IRF5 SLE-risk variants could increase IFN-*α* production in the setting of different antibodies, resulting in high serum IFN-*α* and subsequent SLE risk [83].

The studies published until now have suggested other associations between specific autoantibodies and genetic variants, among which are the associations between anti-RNP and rs1143679 of ITGAM (OR = 1.89), anti-RNP and rs56203834 of TREX1 in European populations (OR = 5.2), anti-Sm and rs7574865 of ITGAM (OR = 0.65), and anti-cardiolipin and rs3099844 of HCP5 (OR = 0.34) [20, 26, 47, 55]. However, all these associations should be confirmed in larger populations and the mechanisms explaining must be identified.

The reduction of C3 and/or C4 serum levels represents a frequent manifestation in patients affected by SLE and could correlate with disease activity [84]. A strong association was well established between homozygous hereditary deficiency of each of the early proteins of the classical pathway of complement activation and SLE development. The deficiency of the C1 complex proteins and of total C4 is recognized as the most prevalent and most severe disease. Indeed, more than 75% of all individuals with deficiency of one of these proteins develop SLE [84]. Conversely, the deficiency of C2 protein seems to be associated with lower prevalence of disease (10%), while C3 deficiency is rarely associated with SLE development, probably due to the rarity of homozygous C3 deficiency [84]. Even though the association between complement proteins deficiency and SLE development has been largely clarified, very few data are available concerning genetic variants associated with C3 and C4 levels reduction, extrapolated by studies not focusing on this specific aspect. A correlation between C4 reduction and the SNP rs33980500 of TRAF3IP2 has been identified in the study conducted by Perricone et al. (OR = 1.96) [63]. Conversely, the reduction of C3 serum level was associated with the genetic variant mir146a rs2910164 (OR = 1.91) [55].

## 9. Age at Disease Diagnosis

The evaluation of the studies published so far identified interesting data concerning the influence of STAT4 genetic variants on age at diagnosis. The SNP rs7574865 of STAT4 resulted in being associated with age at diagnosis lower than 30 years (OR = 1.22) [16, 18]. The frequency of the same genetic variant resulted in being slightly higher in SLE Japanese patients with an age of onset lower than 20 years as compared with patients with age ≥ 20 years, although this difference was not statistically significant [19].

Moreover, rs2233945 of PSORS1C1 resulted in being associated with age at diagnosis. Ciccacci and colleagues observed that patients carrying the variant allele present a lower mean age at disease onset compared with those not carrying the variant (28.6 ± 11.57 years* versus *32.2 ± 11.46 years, *P* = 0.042) [55].

## 10. Chronic Damage

The increase of survival of SLE patients determined the accrual of cumulative organ damage: adverse events of treatment, disease activity, and comorbidities seem to be the major risk factors. The prevention of damage development is a critical issue in the management of SLE patients, as underlined by the recent treat-to-target recommendations [85]. Consequently, the identification of specific biomarkers, able to identify SLE patients with a major risk to develop chronic damage, is an attractive topic. Among the different biomarkers, genetic variants could play a role. The study conducted by Carvalho and colleagues in 2015 suggested a role of VDR polymorphism [86]. The evaluation of 170 Portuguese SLE patients and 192 healthy controls demonstrated an association between different genetic variants and accrual damage. In particular, the frequency of VDR genotypes TaqI TT (rs731236) and Fok I CT (rs2228570) was higher in SLE patients with damage, evaluated by using the SLICC Damage Index (SDI) [86, 87].

The development of osteoporosis with fractures is considered chronic damage in SLE patients and is inserted in the SDI [87]. Bonfá and colleagues performed a case-control study by evaluating 211 premenopausal SLE patients and 154 healthy women, in order to evaluate the association between the RANKL, OPG, and RANK gene polymorphisms and bone parameters. A significantly lower frequency of the RANKL 290 G allele (AG/GG) was identified in the patients with vertebral fractures compared with those without (*P* = 0.011). In the logistic regression analysis, in addition to the age, only RANKL 290A>G remained as an independent risk factor for vertebral fractures in SLE patients [88]. The authors hypothesized that the protection against vertebral fractures that is associated with the AG/GG genotype could be a consequence of decreased osteoclast activation due to RANKL dysfunction or to a local reduction of this molecule in the bone [88].

## 11. Conclusion

**Figure 2:**
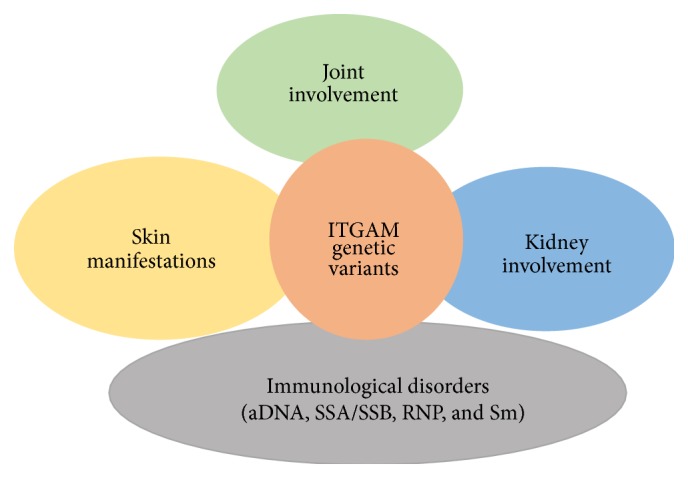
Disease manifestations associated with ITGAM genetic variants.

As widely demonstrated, genetic factors play a pivotal role in SLE pathogenesis. Moreover, in the last years several evidences suggested the role of genetic factors not only in disease susceptibility, but also in the development of specific disease phenotype. Several data are available to date concerning genetic variants involved in renal involvement, while fewer studies have been focused on SLE clinical and immunological manifestations. Interestingly, some genetic variants seem to be involved in the determination of different disease-related manifestations, suggesting a common pathogenetic mechanism, able to identify specific subset of patients. An example of this concept is represented by ITGAM genetic variants that resulted in being simultaneously associated with different disease manifestations (Figure 2). The recent progress leading to the discovery of novel methods to perform genetic studies will definitely allow clearly defining the associations between genes variability and SLE susceptibility and phenotype. Possibly, risk algorithms will be developed permitting a more personalized management of the disease.

However, small populations and lack of all clinical data characterize the studies evaluating the association between genetic and different disease phenotypes, not allowing a sufficient statistical power. Further studies specifically designed to evaluate this issue are needed to clarify the strongest associations.

## Figures and Tables

**Table 1:** Genetic variants associated with disease manifestations.

Disease phenotypes	Genetic variants associated with related SNPs

Skin involvement	ITGAM rs1143679 FCGR2A rs1801274 IL-6 174G/C VDR rs1168268

Serositis	TRAF3IP2 rs33980500, rs13190932, and rs13196377 PTPN2 rs2542151

Kidney involvement	HLADR2, HLADR3 rs2187668 STAT4 rs7574865, rs11889341, rs7568275, and rs7582694 ITGAM rs1143683, rs1143679 IRF5 rs2004640, rs2079197, and rs10488631 IRF7 rs4963128 TNFS4 rs2205960 DNAse I Q222R

Neurologic disorder	TREX1 rs922075, rs6776700, rs6442123, rs2242150, and rs11797

Joint involvement	ITGAM rs1143679 FGCR2A, FCGR3A VDR rs3890733 Mir146a rs2910164

Hematological features	IL-21 rs907715 STK17A haplotype TAGTC

Immunologic disorders	Anti-dsDNA HLADR2, HLADR3 rs2187668 STAT4 rs7582694, rs7574865 ITGAM rs1143679, rs9888739 IRF5 rsrs10488631 SSA/SSB ITGAM rs1143679 IRF7 rs4963128 HCP5 rs3099855 HLADR3 rs2187668 RNP ITGAM rs1143679 Sm ITGAM rs7574865 aCL HCP5 rs3099844 C3 reduction Mir146a rs2910164 C4 reduction TRAF3IP2 rs33980500
